# An exceptional challenging case: Anterior shoulder dislocation with ipsilateral humeral shaft fracture complicated with an upper extremity compartment syndrome

**DOI:** 10.1016/j.ijscr.2023.108237

**Published:** 2023-04-19

**Authors:** Abdellatif Benabbouha, Youssef Benyass, Hicham Sallahi, Omar Margad

**Affiliations:** Department of Orthopaedics, Military Training Hospital Avicenne, University Cadi Ayyad, BP 40150 Marrakech, Morocco

**Keywords:** Shoulder dislocation, Humeral shaft fracture, Acute compartment syndrome, Simultaneous

## Abstract

**Introduction:**

Combination of shoulder dislocation with ipsilateral shaft humeral fracture is extremely rare and the occurrence of upper extremity compartment syndrome as a complication is even rarer.

**Case presentation:**

A 36-year-old male, sustained a road traffic accident as a pedestrian struck by a vehicle. He was diagnosed with an anterior dislocation of the right shoulder with an ipsilateral open transverse fracture of the middle third of the humeral shaft. He was treated with closed reduction and Hackethal bundle nailing. The next day, the patient developed acute compartment syndrome and underwent multiple fasciotomy.

**Discussion:**

This particular combination represents a great surgical challenge in orthopedics and there is no clear consensus until now regarding its management. We review the mechanism and the appropriate treatment of this injury.

**Conclusion:**

We think it is critical to emphasize the original character of our case, because it is probably the first report to describe this special injury associated with upper extremity compartment syndrome.

## Highlights

• Combination of shoulder dislocation with ipsilateral shaft humeral fracture is extremely rare
• The mechanism of this combination is not well understood until now
• This special injury represents a surgical challenge in orthopedics and there is no clear consensus until now regarding its management.

## 1 Introduction

Traumatic shoulder dislocation is one of the most common reasons for emergency room visits. It is usually associated with other injuries, especially greater tuberosity fractures and rotator cuff tears [1]. Nevertheless, combined shoulder dislocation and ipsilateral humeral shaft fracture is extremely rare. Furthermore, this particular combination represents a great surgical challenge in orthopedics.

We present an even rarer combination, has never been reported before in the literature where there was simultaneously a shoulder dislocation and an ipsilateral humeral shaft fracture that complicated by upper extremity compartment syndrome. This case is reported in line with the SCARE criteria [2].

## 2 Case presentation

**Fig. 1:**
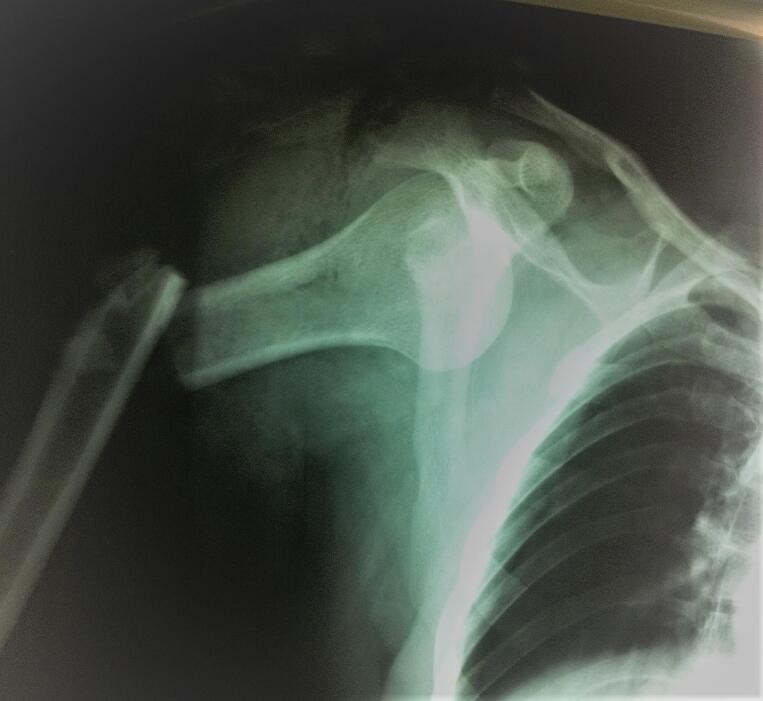
X-ray showing anterior dislocation of the right shoulder with ipsilateral shaft humeral fracture.

**Fig. 2:**
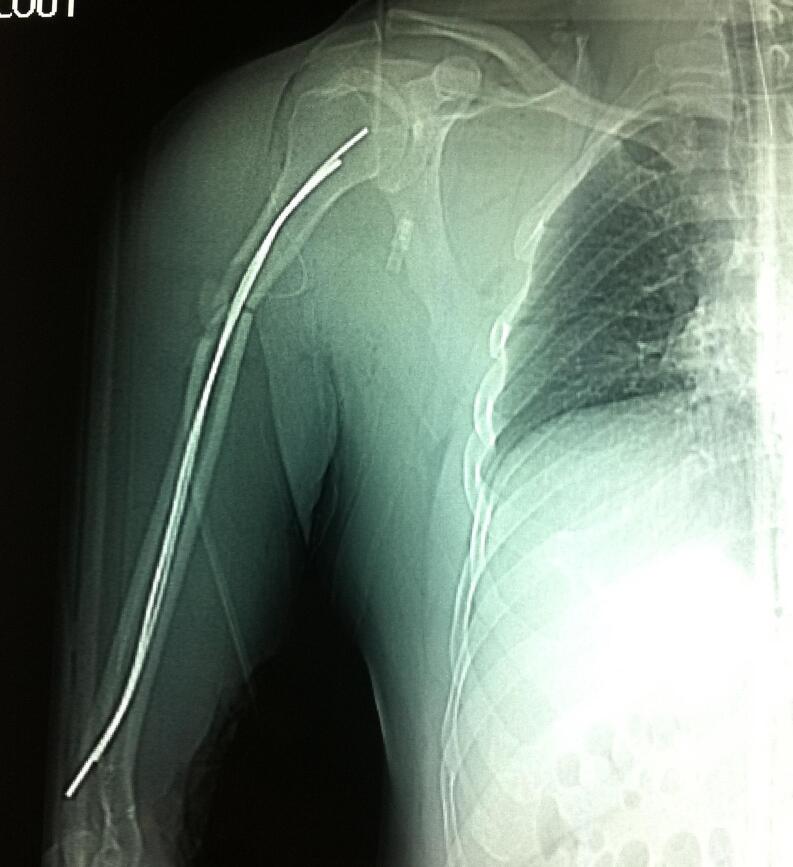
Post-operative X-rays after reduction and Hackethal bundle nailing.

**Fig. 3:**
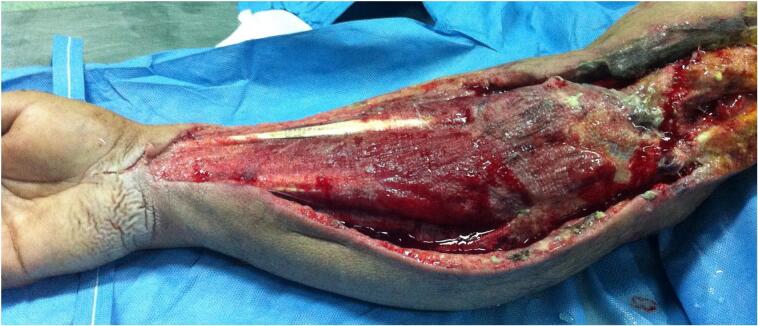
A lazy S fasciotomy of the anterior aspect of the forearm and arm.

**Fig. 4:**
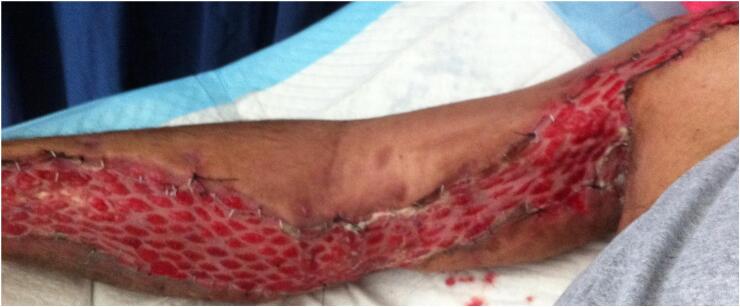
Skin grafting of the anterior aspect of the forearm and arm.

A 36-year-old male, right-handed, with no prior medical history, sustained a road traffic accident as a pedestrian struck by a vehicle. He was injured on the right side of his body. He was admitted immediately to our hospital. Upon arrival, he was conscious and hemodynamically stable. Clinical examination showed a visible deformity of his right arm with diffuse traumatic ecchymosis. There was pain, tenderness and an open wound over anterior aspect of the right shoulder measuring 5 cm with moderate soft tissue injury corresponding to grade II of the Gustilo classification. There was no distal neurovascular deficit. Radiographs indicated an anterior dislocation of the shoulder with ipsilateral transverse fracture of the middle third of the shaft humerus (Fig. 1). Thus, the patient was urgently carried to the operating theatre. Under general anesthesia closed reduction of the shoulder dislocation was performed manually without difficulty. Then, the humeral shaft fracture was stabilized using Hackethal's bundle nailing after debridement of the wound (Fig. 2). The right limb was immobilized in a plaster slab. The next day, the patient developed severe pain and swelling in the right arm and forearm, associated with paresthesia and loss of sensation in the hand. He was unable to flex all fingers. Furthermore, radial and ulnar pulses were present but weak. Therefore, the diagnosis of compartment syndrome was clinically obvious without measuring compartment pressures. The patient was returned to the operating room for decompression. Indeed, we performed a lazy S fasciotomy of the anterior aspect of the forearm and arm. The superficial fascia was released and the deep fascia was opened (Fig. 3). Postoperatively, there was no drainage system and the limb was immobilized with a plaster splint. The pain was relieved after the fasciotomy, the neurologic functions were recovered, and the swelling was subsided. Whereas, the primary closure of the wounds was impossible after repeat surgical debridement with removal of devitalized tissues. Hence, skin grafting was performed on 10th day (Fig. 4). The upper limb was immobilized for 3 weeks while the wound healed. A passive rehabilitation was undertaken. At 4-month follow-up, the bundle nailing was removed and radiographs revealed good fracture union. At the latest follow-up of 10 months, he had a useful range of motion compared with the contralateral limb with a slight restriction in elbow flexion. Additionally, the Constant score was approximately 80/100 and the patient had returned to normal activities.

## 3 Discussion

Although isolated shoulder dislocations or humerus fractures are common injuries, it is extremely rare for both injuries to occur simultaneously in the same limb. Since Winderman's first description in 1940 [3], only a few cases have been documented in the orthopedic literature [4], [5], [6], [7], [8], [9], [10], [11]. Thus, our case is probably the first to describe this special injury associated with upper extremity compartment syndrome.

To date, the mechanism of this combination is not well understood. Various hypotheses have been proposed in the literature. It still remains unclear whether fracture or dislocation is the first to occur. Some authors assume that the energy of the trauma was attributed to the humeral shaft and shoulder, causing the dislocation and fracture simultaneously [9], [10]. In contrast, others propose that indirect forces result in shoulder dislocation first, followed by direct forces which cause the fracture [7], [11]. Based on our interpretation, the probable mechanism of our case was transmitting force along the shaft to the shoulder while the shoulder was extended, abducted with the hand outstretched, resulting in anterior dislocation and transverse fracture.

Given the rarity of this complex injury, there are no sufficient orthopedic reports concerning its management, and there is no clear consensus until now. According to literature reviews, some authors recommend an early closed reduction of the shoulder, sometimes aided by a Steinman pin [3], [12]. However, others prefer to fix the humeral fracture first to facilitate shoulder reduction, and there are several methods reported: plating, intramedullary nailing, bundle nailing, and external fixation [9], [13], [14], [15]. As an alternative, four reported cases were conservatively treated with a functional splint, which led to successful outcomes [3], [7], [16].

In our case, the management was severely affected by the occurrence of acute compartment syndrome. In fact, this serious complication is characterized by an increase in pressure within a closed myofascial space leading to tissue ischemia. As is well known, this surgical emergency can occur due to several causes, including fractures, crush syndrome, arterial injuries, infections, etc. [17], [18]. Typically, it is diagnosed using the 5P rule which includes pain, paresis, pallor, paresthesias and pulseless arteries [19]. It seems necessary to measure intracompartmental pressure when clinical presentation is not obvious. Hence, immediate fasciotomy should be carried out urgently and any delay in diagnosis or treatment leads to irreversible ischemic lesions that threaten the functional prognosis of the limb [17], [18].

## 4 Conclusion

Although the diagnosis and potential threat to limbs of acute compartment syndrome is well known to most clinicians, we still see cases of delayed or missed diagnosis in our practice. Therefore, physicians need to be aware of the seriousness of acute compartment syndrome and its consequences on both the patient and the physician.

Lastly, we think it is critical to emphasize the original character of the case described. To our knowledge, this specific combination involving simultaneously a shoulder dislocation and an ipsilateral humeral shaft fracture that was complicated by upper extremity compartment syndrome has never been reported before in the literature.

## Consent

Written informed consent was obtained from the patient for publication of this case report and accompanying images. A copy of the written consent is available for review by the Editor-in-Chief of this journal on request.

## Ethical approval

This study is exempt from ethnical approval.

## Funding

There is no founding source.

## Author contribution

‐ The patient had been operated on by Pr. Abdellatif Benabbouha and Pr. Omar Margad.
‐ The bibliographic research was done by Pr. Youssef Benyass and Pr. Hicham Sallahi.
‐ The article was written by Pr. Abdellatif Benabbouha.

## Guarantor

Pr. Abdellatif Benabbouha.

## Research registration number

Not applicable.

## Declaration of competing interest

Authors report no conflicts of interest.

## References

[1] Zacchilli M.A., Owens B.D. (2010). Epidemiology of shoulder dislocations presenting to emergency departments in the United States. J. Bone Joint Surg. Am..

[2] Agha R.A., Franchi T., Sohrabi C., Mathew G., Kerwan A., S.C.A.R.E. Group (2020). The SCARE 2020 guideline: updating consensus Surgical CAse REport (SCARE) guidelines. Int. J. Surg..

[3] Winderman A. (1940). Dislocation of the shoulder with fracture of the shaft of the humerus. Bull. Hosp.Jt. Dis. Orthop. Inst..

[4] Flint J.H., Carlyle L.M., Christiansen C.C., Nepola J.V. (2009). Case report and literature review anterior shoulder dislocation with three-part proximal humerus fracture and humeral shaft fracture. Iowa Orthop. J..

[5] Chen C.H., Lai P.L., Niu C.C., Chen W.J., Shih C.H. (1998). Simultaneous anterior dislocation of shoulder and fracture of the ipsilateral humeral shaft. Int.Orthop..

[6] Chirputkar K., Basappa P., McLean I., Nimon G. (2006). Posterior dislocation of the shoulder with ipsilateral humeral shaft fracture: a case report and review of literature. Acta Orthop. Belg..

[7] Kontakis G.M., Galankis I.A., Steripoulous K.A. (1995). Dislocation of the shoulder and ipsilateral fracture of the humeral shaft: case reports and literature review. J. Trauma.

[8] Kapila R., Awasthi B., Sehgal B., Sharma A., Kapila P. (2018). Anterior glenohumeral dislocation with ipsilateral shaft humerus fracture - a rare co-occurrence; case report from Hills of North India. J. Orthop. Case Rep..

[9] Sankaran-Kutty M., Sadat-Ali M. (1989). Dislocation of the shoulder with ipsilateral humeral shaft fracture. Arch. Orthop. Trauma Surg..

[10] Karimi-Nasab M.H., Shayesteh-Azar M., Sajjadi-Saravi M., Mehdi Daneshpoor S.M. (2012). Anterior shoulder dislocation and ipsilateral humeral shaft fracture. IranJ. Med. Sci..

[11] Canosa I., Areste J. (1994). Dislocation of the shoulder with ipsilateral humeral shaft fracture. Arch. Orthop. Trauma Surg..

[12] Barquet A., Schimchak M., Carreras O. (1985). Dislocation of the shoulder with the fracture of ipsilateral shaft of humerus. Injury.

[13] Lyu F., Wang H.X., Bi C., Shen S.M., Wang Q.G., Wu X.M. (2020). Management of dislocation of the shoulder joint with ipsilateral humeral shaft fracture: initial experience. Orthop. Surg..

[14] Gupta Y., Jha R.K. (2015). Compound fracture of humeral shaft associated with two-part fracture dislocation of ipsilateral shoulder: a rare combination. J. Surg. Case Rep..

[15] Farooque K., Khatri K., Dev C., Sharma V., Gupta B. (2014). Mechanism of injury and management in traumatic anterior shoulder dislocation with concomitant humeralshaft and ipsilateral scapula fracture: a case report and review of the literature. J. Med. Case Rep..

[16] Davick J., Zadalis R., Garvin K. (1995). Anterior glenohumeral dislocation with ipsilateral humeral shaft fracture. Orthopedics.

[17] Schmidt A.H. (2017). Acute compartment syndrome. Injury.

[18] Prasarn M.L., Ouellett E.A. (2011). Acute compartment syndrome of the upper extremity. J. Am. Acad. Orthop. Surg..

[19] Hanandeh A., Mani V.R., Bauer P., Ramcharan A., Donaldson B. (2019). Identification and surgical management of upper arm and forearm compartment syndrome. Cureus.

